# Clotting Dysfunction in Sepsis: A Role for ROS and Potential for Therapeutic Intervention

**DOI:** 10.3390/antiox11010088

**Published:** 2021-12-30

**Authors:** Maria Elisa Lopes-Pires, Jéssica Oliveira Frade-Guanaes, Gregory J. Quinlan

**Affiliations:** 1 National Heart and Lung Institute, Faculty of Medicine, Imperial College London, London W12 0NN, UK; g.quinlan@imperial.ac.uk; 2 Adventist University of Sao Paulo, Engenheiro Coelho, Sao Paulo 05858-001, Brazil; jessica.guanaes@unasp.edu.br

**Keywords:** sepsis, oxidative stress, nitric oxide, netosis, platelets, clotting dysfunction, vascular endothelium dysfunction

## Abstract

Sepsis is regarded as one of the main causes of death among the critically ill. Pathogen infection results in a host-mediated pro-inflammatory response to fight infection; as part of this response, significant endogenous reactive oxygen (ROS) and nitrogen species (RNS) production occurs, instigated by a variety of sources, including activated inflammatory cells, such as neutrophils, platelets, and cells from the vascular endothelium. Inflammation can become an inappropriate self-sustaining and expansive process, resulting in sepsis. Patients with sepsis often exhibit loss of aspects of normal vascular homeostatic control, resulting in abnormal coagulation events and the development of disseminated intravascular coagulation. Diagnosis and treatment of sepsis remain a significant challenge for healthcare providers globally. Targeting the drivers of excessive oxidative/nitrosative stress using antioxidant treatments might be a therapeutic option. This review focuses on the association between excessive oxidative/nitrosative stress, a common feature in sepsis, and loss of homeostatic control at the level of the vasculature. The literature relating to potential antioxidants is also described.

## 1. Introduction

Sepsis is a life-threatening condition that affects 30 million people worldwide per year and is considered one of the main causes of death amongst critically ill patients. Seen in context, sepsis-related mortality from 2009 to 2019 averaged 33.7% in North America, 32.5% in Europe, and 26.4% in Australia [1]. In the United Kingdom, around 250,000 cases and 44,000 deaths from sepsis are reported every year [2]. In the United States, similarly high rates of sepsis diagnosis are reported annually, at some 1.7 million cases, with mortality rates in the region of 270,000 [3]. Specialist treatment for patients with sepsis often requires intensive care support to maintain failing organs systems, including the lungs, heart, and kidneys; such treatments can be complex and may require an extended length of hospital stay, all of which places a considerable fiscal burden on healthcare providers. In the United Kingdom, around two-thirds of patients with sepsis are treated in intensive care units (ICUs), with an annual estimated cost at £15.6 billion [4]. This represents a significant component of annual healthcare budgets; moreover, the need for further support for recovering patients as provided by primary care providers places an even greater burden budget. Again, for example, in 2011, U.S. hospitals spent $24 billion treating patients with sepsis, representing 13% of total health care costs (reviewed by [5]).

Defining criteria for sepsis and associated syndromes have evolved over the years; the current definition describes sepsis as an uncontrolled host-mediated response to infection and life-threatening organ dysfunction. Any patient is diagnosed with sepsis when they attain a score of 2 by sequential organ failure assessment (SOFA). SOFA comprises a scoring system based around the functionality of respiratory, hepatic, cardiovascular, central nervous and renal systems, and platelet count [6,7].

In response to pathogen infection, a protective pro-inflammatory response is initiated, but this can become deleterious and of extended duration, leading to sepsis, and over activation of the inflammatory system results in the production of reactive oxygen (ROS) and nitrogen species (RNS) to the extent that endogenous antioxidant protection becomes overwhelmed. The consequences of this are diverse in nature, including impacts on redox-based cell signalling systems; direct damage to biomolecules; and, perversely, the potential for immunosuppression. A common component of severe sepsis is endothelial damage and the triggering of the coagulation system, which can progress to DIC, which is marked by macro and microvascular thrombosis and hemorrhage and is a leading cause of organ damage in sepsis [8]. This review aims to explore the potential role for ROS and RNS as initiators for adverse clotting and bleeding events in sepsis. Efforts to target these processes using antioxidant therapies will also be discussed.

## 2. Disseminated Intravascular Coagulation in Sepsis 

**Figure 1:**
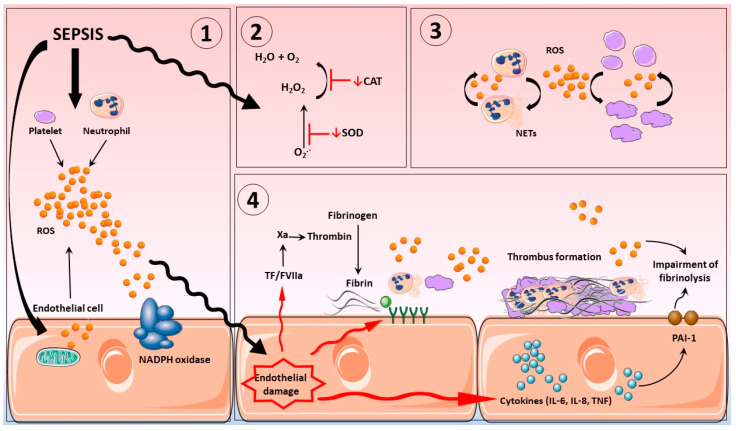
Sepsis induces oxidative stress and disseminates intravascular coagulation. (**1**). Sepsis induces ROS release by platelets, neutrophils, and endothelial cells. The majority of excessive ROS production is generated by mitochondria and NADPH oxidase present in endothelial cells, platelet, and neutrophil. (**2**). The overproduction of ROS results in depletion of endogenous antioxidant systems, including but not limited to SOD and catalase. (**3**). ROS release from activated inflammatory cells such as neutrophils and platelets further propagate inflammatory responses including further ROS production, processes that are self-sustaining and ever expanding. (**4**). Damage to the vascular endothelium augments inflammatory cytokine production via ROS-mediated stress responses and activates the coagulation system and expression of adhesion molecules, all of which results in elevation of fibrin deposition; impairment of fibrinolysis; and, consequently, thrombus formation. ROS: reactive oxygen species. CAT: catalase. SOD: superoxide dismutase. TF: tissue factor. NETs: neutrophil extracellular traps.

To maintain homeostatic control of blood flow through the circulation, it is essential that the processes of platelet plug formation and fibrinolysis are tightly regulated. If coagulation or anticoagulation mechanisms become dysfunctional, loss of homeostatic control can result in pathologies such as DIC, which is commonly encountered in patients with sepsis. DIC is a coagulopathy that occurs as a result of extensive and inappropriate activation of the coagulation system. Persistent coagulation results in thrombotic occlusion of small- and medium-sized blood vessels of the circulation via the establishment of microthrombi owing to fibrin formation (reviewed by [9]). Additionally, cessation of fibrinolysis, the mechanism responsible for lyzing the clot generated by activation of hemostatic pathways, contributes to DIC development in sepsis (reviewed by [10]). It has been reported that the levels of plasminogen activator inhibitor-1 (PAI-1), the protein responsible for assuring the clot preservation, is elevated in sepsis and is correlated with cytokines’ releases and poor patients’ outcome [11,12,13]. Another study, with the plasma of diabetics patients, demonstrated a strong correlation between the rise in oxidation markers (oxidized low-density lipoprotein and nitrotyrosine) and the impairment of the fibrinolysis process [14] (see Figure 1).

As such, compromised blood flow to key organs can result in multiple organ failure (MOF) and mortality, although other hemodynamic and metabolic disorders can similarly disrupt blood flow with similar outcomes [15,16]. In this regard, clinical guidelines relating to DIC state that, for better treatment and improved patient outcome, it is important to differentiate specific clinic phenotypes of disease type, including (1) increased fibrinolysis, such as would be associated with leukemia, trauma, and aortic or obstetric diseases; (2) suppressed fibrinolysis, associated with organ failure and septicemia; or (3) balanced fibrinolysis, such as would be observed with solid cancers [17,18]. Up to 40% of patients with sepsis present with or develop DIC [5,19,20,21]. During episodes of DIC, both bleeding and clotting events occur concurrently, which poses considerable issues regarding therapeutic approaches given the need to try and balance these opposing events [22].

A variety of clinical scoring methods have been developed with the intent to help physicians estimate the severity of disease in sepsis; however, such systems lack precision as definitive symptoms and clear diagnosis criteria are not obvious in many patients [16,23]. Currently, the most widely used DIC criteria scores are those as set out by The International Society of Thrombosis and Haemostasis (ISTH) and the Japanese Association for Acute Medicine (JAAM) [16].

The development of DIC in sepsis is thought to involve crosstalk between the inflammatory system activation and the overstimulation of coagulation and, more specifically, a deficient natural anticoagulant system, which leads to platelet activation and neutrophil extracellular trap formation, followed by fibrin deposition [24,25,26].

The disproportionate host inflammatory response to pathogens during sepsis results in dysfunction and damage to the vascular endothelium, largely due to the effects of a cytokine storm, with inflammatory cytokines such as tumor necrosis factor a (TNFα), interleukins 1b and 6 (IL-1b and IL-6), and interferon-gamma (IFNg) being implicated [27]. IL-6 is reported to play a central role in the activation of coagulation by tissue factor (TF) [16]. TF is a transmembrane glycoprotein able to activate the coagulation cascade when it is exposed to blood, under which circumstances the formation of a complex with FVII/FVIIa can ensue (reviewed by [28]), a frequent occurrence during endotoxemia [29]. Established beliefs indicated that TF was present and active only on general cell membranes; however, recent evidence has demonstrated the presence of TF on the surface of extracellular vesicles and microparticles (MPs), derived from platelets, leukocyte, and endothelial cells in patients with sepsis and DIC [30]. In addition to TF releasing, MPs also induce exposure of phosphatidylserine (PS), a phospholipid found on the surface of activated platelets that binds to an array of intrinsic and extrinsic factors to generate thrombin, a fundamental event of blood coagulation [31]. In addition, PS is associated with a significant increase in IL-6 and platelet activity, which correlates with the progression of endothelial damage and leukocyte activation [32,33].

While overexpression of pro-inflammatory cytokines enhances the activation of clotting cascades in sepsis, there is also good evidence to indicate associated impairment of pathways for essential natural anticoagulant activity, such as the antithrombin system, which is an important inhibitor of thrombin formation and FXa activation; the protein C system including protein S, which is an essential co-factor for the activity of protein C; and for thrombomodulin expression on endothelial cells (reviewed by [28]). Levels of protein C and antithrombin are markedly reduced in patients with sepsis with DIC [19]. Moreover, higher levels of protein C are associated with better patient outcomes in general, and as such may offer use as a both a clinical biomarker and a therapeutic target [34].

## 3. Vascular Haemostasis in Sepsis

### 3.1. Endothelium

The glycocalyx is a complex structure that consists of proteoglycans, glycoproteins, and glycosaminoglycan chains found on the surface of endothelial cells, and it plays a critical role in the regulation of vascular permeability; for a full description, see [35]. Sepsis-induced endothelial dysfunction including glycocalyx shedding results in increased leucocyte adhesion to endothelial cells, thereby exacerbating tissue damage [36]. Injury to the glycocalyx is known to be accentuated by increased levels of inflammatory molecules such as IL-1b. Diabetes likely further increases the associated risk; to this end, a recent study undertaken in mice demonstrated that diabetes limits glycocalyx synthesis, which is further damaged by endotoxemia [37]. Importantly, patients with diabetes have higher hospital admission rates when compared with non-diabetics [38].

It is increasingly recognized that disruption of vascular endothelium functionality is an important contributing factor to the onset of the progression of sepsis, including coagulation disorders, where TF and other procoagulant factors are known to be increased by high levels of inflammatory mediators. As such, studies have shown significant associations between markers of endothelial dysfunction and mortality, including increases in expression of endocan, Ang-2 and HMGB-1, and decreasing levels of protein C with TF levels [34].

Given the emerging understanding of endothelial dysfunction in sepsis, therapeutic approaches designed to protect the endothelium and glycocalyx are the subject of ongoing investigation. Indeed, a recent study has demonstrated in an animal rodent model of sepsis that recombinant antithrombin was able to protect the endothelial glycocalyx from injury, thus maintaining vascular integrity. Moreover, this approach was shown to decrease levels of syndecan-1, which is an important biomarker of glycocalyx damage [39]. Moreover, outcomes of ProCESS, a randomized study for resuscitation strategies undertaken in 1341 patients, found associations for the expression of endothelial biomarkers of permeability with mortality in sepsis, including at baseline and 24 h mortality. A decrease in expression of VEGF was observed, whereas there was an increase in the expression of angiopoietin-2, tissue plasminogen activator, thrombomodulin, and von Willebrand factor [40], leading to an increase in thrombus and associated with the increase in mortality in sepsis.

### 3.2. Platelets

Platelets are anucleate blood cells derived from megakaryocytes that are able to release cytokines, and that interact with leukocytes and endothelial cells, performing fundamental roles in both vascular homeostasis and coagulation. Platelet activation represents an important host response to infection for both innate and adaptive immunity [41]. In sepsis, platelets are implicated in coagulation dysfunction, through activation of pro-inflammatory mediators such as platelet activating factor and increasing fibrin formation via the expression of procoagulant molecules, including P-selectin [28,42,43]. However, decreased platelet counts (thrombocytopenia) may act as a predictor of mortality for patients with sepsis/septic shock and DIC [44]. The reasons for persistent thrombocytopenia in sepsis are not fully understood, but some theories suggest that this may be due to reduced platelet production, enhanced turnover, or spontaneous aggregation of platelets and enhanced platelet consumption through the formation of microthrombi. Although persistent platelet activation is most often related to septicemia, a few studies have shown that platelet aggregation is decreased in experimental sepsis [45], possibly signalled via the TNF pathway [46]. Moreover, the reduction in platelet aggregation seen in patients with sepsis is more pronounced depending on the severity sepsis, stage of disease, and the presence of DIC [47].

### 3.3. Neutrophils

Neutrophils are white blood cells that play a critical role in the immune response (reviewed by [48]) as well as in sepsis, which is associated with an excessive activation of neutrophils. Indeed, such neutrophil overstimulation is understood to be a key contributor to manifestations of sepsis and associated syndromes. In addition to the production of oxidants including hypochlorous acid (HOCL), hydrogen peroxide (H_2_O_2_) and superoxide (O_2_^•−^), proteases, and chemokines, activated neutrophils are also capable of undergoing a process named netosis. The release of neutrophil extracellular traps (NETs) during netosis occurs when nuclear DNA decondenses to release web-like structures of linear DNA from the cell that are interlaced with histones, myeloperoxidase, and other antimicrobial peptides such as elastase [49,50,51]. NETs are able to trap and kill microorganisms owing to the activity of associated antimicrobial proteins, and they also limit parasite dissemination [52]. However, unwanted collateral effects linked to netosis have also been described, including the induction of tissue injury mediated by extracellular histones and granular proteins [53,54]. Moreover, some substances, such as elastase and myeloperoxidase, released by NETs are considered to be damage-associated molecular patterns (DAMPs) and can cause tissue injury through activation of toll-like receptors on endothelial cells, leading to dysfunction [55,56,57]; additionally, extracellular MPO is still capable of forming the damaging oxidant HOCL. Interestingly, it has been shown that the inhibition of neutrophil elastase can prevent NETs’ formation and reduces septic shock in animal models, and thus may offer a therapeutic target for septicemia [58].

Some recent literature has demonstrated an association with the severity of sepsis and levels of NETs’ formation. In this regard, higher levels of NETs’ production correlated with the worsening and severity of sepsis and organ failure in humans; moreover, NETs’ formation during the initial stages of sepsis was also positively correlated with levels of key inflammatory cytokines IL-8, IFN-gamma, and TNFα [59]. Furthermore, inflammatory modulation by NETs was reported lead to severe damage in the liver, spleen, and kidneys in a murine model, processes that were abrogated by the use therapeutic of anti-citrullinated protein antibody, a NET formation inhibitor [57,60,61]. Other modulators released during the acute inflammatory response have also been linked to the induction of NETosis. For example, cold-inducible RNA-binding protein (CIRP), which is a DAMP, is known to be associated with organ injury and increased mortality in sepsis, and has recently been shown to enhance NETosis in the mice lungs during sepsis in an animal model induced by a cecal ligation and puncture (CLP) model [62]. Previous studies have also demonstrated that some antibiotics such as fluoroquinolones, macrolides, and a few b-lactams are capable of modulating the formation of NETs and, as such, may offer a protective role in early sepsis, this being ascribed to an immunomodulatory function possibly owing to downregulation of the PKC-Akt-mTOR pathway [50].

In addition, given the context of this review, NETs’ release is further linked to the evolution of DIC via the reduction in the levels of antithrombin and, as such, may provide a possible therapeutic target to treat DIC [58,63].

Importantly, the DNA molecule contains a polyphosphate backbone and is a known intracellular storage polymer of phosphate. Persistence of NETs is normally regulated by plasma DNAase 1 activity; there are nevertheless circumstances when such control is lost or overwhelmed, and net formation predominates [64]. DNA provides a negatively charged surface for the autocatalytic activation of Factor XII and the intrinsic pathway of coagulation, leading to increased thrombin generation and risk of thrombosis. Moreover, histones released with DNA are potent platelet activators, causing platelet degranulation and release of polyphosphate (PolyP). PolyP has been shown to be a highly potent activator of the contact pathway in vitro and in vivo [65,66], binding with high affinity to several of its protein components. Therefore, targeted inhibition of the Factor XII pathway may offer a therapeutic option in sepsis.

## 4. Oxidative and Nitrosative Stress

Reactive oxygen species (ROS) are chemical species encompassing free radicals and related oxygen containing species. The most encountered inorganic ROS include the following: the superoxide radical anion (O_2_^•−^), the hydroxyl radical (^•^OH), hydrogen peroxide (H_2_O_2_), and hypochlorous acid (HOCl) [67,68,69]. Most ROS production is purposeful, allowing for utilizations of oxygen for aerobic metabolism and biosynthesis. These processes are highly regulated to limit any adverse consequences related to the inappropriate activation of oxygen to more reactive forms. Should this control be undermined or lost, excessive ROS production can affect redox-regulated cell-signalling responses, leading to aberrant stress responses, as seen in sepsis. To this end, over production of O_2_^•−^ and H_2_O_2_, both key signalling species, can lead to redox-regulated pro-inflammatory transcription factor over activation as well as significant and inappropriate pro-inflammatory responses. The production of directly damaging ROS including ^•^OH and HOCl can also result in damage and dysfunction to an array of biomolecules, including lipids, proteins, and nucleic acids, as well as the production of toxic end-products such as aldehydes and protein carbonyls. Moreover, interactions between relatively innocuous species such as O_2_^•−^ and nitric oxide (NO) in equimolar proportions result in the production of peroxynitrite (ONOO^−^), which, although not a free radical, is nevertheless a very aggressive species capable of inflicting damage similar to that ascribed to ^•^OH. Furthermore, ONOO^−^ is capable of reacting with proteins and peptides, thereby causing functional changes; modifications include s-nitrosylation, glutathionylation, and tyrosine nitration. NO and ONOO^−^ are described as reactive nitrogen species (RNS); other RNS include nitroxyl (HNO), nitrosonium cation (NO^+^), S-nitrosothiols (RSNOs), NO_2_^−^ (nitrite), and dinitrosyl iron complexes, excluding NO_3_ [70,71]. Under normal conditions, endothelial cells generate NO through eNOS, influencing cGMP levels and relaxing vascular smooth muscle, thereby promoting the vessel dilation. NO is also a known inhibitor of platelet activity through the sGC-cGMP-PKG pathway, following different mechanisms: (i) PKG reduces intraplatelet Ca^2+^ levels inhibiting platelet shape change and, consequently, inhibiting the release of mediators involved in platelet aggregation; (ii) PKG promotes the phosphorylation of TXA_2_ receptor, suppressing the effects of the platelet agonist; (iii) platelet aggregation is inhibited by the synergic effect of cGMP and cAMP; and (iv) cGMP inhibits PI3K, which is responsible for the activation of integrin αIIbβ3, a transmembrane glycoprotein signalling receptor essential for normal platelet function. NO donors also have been shown to inhibit platelet aggregation independently of sGC [72].

Additionally, it is well reported that haemoglobin (Hb) is capable of binding to NO and its metabolites. S-nitrosothiols and dinitrosyl iron complexes bind to the heme in Hb, working as an NO store, preventing NO oxidation. Moreover, despite the fact that endothelial NO synthesis in venous circulation is disabled because of a low concentration of oxygen, the Hb stores of NO guarantee that NO is available to the venous circulation [73,74]. Appropriate regulation of NO generation and distribution is thus fundamental for the maintenance of vascular tone and normal blood flow, regulating platelet and leukocyte adhesion to endothelium and, ultimately, the distribution of oxygen and nutrients to the body [75,76,77]. Additionally, heme is converted to carbon monoxide (CO), free iron, and bilirubin through heme oxygenase (HO-1) action. Although CO has been reported to induce anti-inflammatory cytokines and downregulate pro-inflammatory cytokines release (reviewed by [78]), its pro-coagulant and anti-fibrinolysis activities (reviewed by [79]) might be substantially involved in the induction of generalized coagulation in sepsis.

Normally, the collateral effects of ROS production are limited because of an armory of protective strategies chiefly facilitated by diverse forms of antioxidant protection and efficient removal and repair mechanisms. However, under circumstances such as excessive inflammation, traumatic injury, and cell/tissue ischaemia, excessive levels of ROS formation can occur to the extent that endogenous protection becomes overwhelmed, a scenario often observed in the critically ill, and particularly so during sepsis. Indeed, there is well-established literature demonstrating oxidative and nitroasive modification/damage of biomolecules in the critically ill [80,81,82,83,84]. In addition, the production of DAMPs (damage-associated molecular patterns) and PAMPs (pathogen associated molecular patterns) and subsequent binding to activation of TLRs (toll like receptors) induce increased ROS release by an array of cells, including endothelial cells, platelets, and neutrophils [85]. Such activation further promotes additional ROS production by these cells, creating a self-sustaining and ever-expanding ROS activation system, which further negatively impacts patient clinical presentation [86,87,88,89].

As for the systems that generate ROS in sepsis, these are complex, including NADPH oxidase (NOX) and dual oxidase enzymes (DuOX); mitochondrial respiration and dysfunction; the activities of cyclooxygenases and lipoxygenases; xanthine oxidoreductases (XOR); the effects of ischaemia reperfusion injury; NO production by NOS enzymes; and loss of homeostatic control for iron recycling, allowing for production of directly damaging ROS. See Table 1 for specific details.

## 5. The Role of Oxidative and Nitrosative Stress in Sepsis-Related Haemostasis

### 5.1. Glycocalyx

The glycocalyx of the endothelium is an intravascular lubricant layer, composed of membrane-binding domains and plasma proteins that separate circulating blood from vessel walls. It is a major contributor to cardiovascular homeostasis, controlling thrombus development, vascular permeability, together with the provision of anti-inflammatory and antioxidant defenses [102,103]. Severe inflammation promotes glycocalyx shedding, altering structure, and compromising function [102]. Oxidative stress is thought to be a major contributor for this impairment, which ultimately leads to the synthesis and exposure of adhesion molecules and, subsequently, the influx of leukocytes and platelets to the vascular bed [104,105]. Indeed, it has been reported that, in diseases where oxidative stress plays an important role, such as sepsis or post-cardiac arrest syndrome, shedding of glycocalyx structures was apparent (see Figure 1) [106].

#### 5.1.1. Mitochondria

Mitochondria are the principal site of ROS generation in endothelial cells both in health and during sepsis [107], and numerous studies have reported the impacts of altered mitochondrial activity on the function of blood vessel. For instance, in human coronary arterioles, rotenone and myxotgiazol, inhibitors of mitochondrial complex I and III, respectively, mitigated flow-induced dilatation associated with O_2_^•−^ and H_2_O_2_ release, whereas apocynin, an NADPH oxidase inhibitor, did not affect the increase in ROS generation induced by shear stress [108]. Additionally, Lowes et al. showed that human umbilical vein endothelial cell incubated with LPS; promoted elevated ROS generation; lowered mitochondrial membrane potential; and increased the release of cytokines IL-1b, IL-6, IL-8, and IL-10. These effects were all abrogated by MitoQ, a mitochondrial targeted antioxidant [109]. MitoTEMPO, another mitochondrial superoxide scavenger, also ameliorated organ dysfunction and improved the survival rates in a murine model of sepsis involving the CLP model [110]. Additionally, the antioxidant protein paraoxonase-2 (PON2) is involved in the control of oxidative stress, reduction of inflammation, and protection against atherosclerosis. Furthermore, Altenhöfer et al. have demonstrated that PON2 decreased O_2_^•−^ generation from the endothelial cells of human mitochondrial complex I and complex III at the inner mitochondrial membrane, supposedly by acting on coenzyme Q10 (CoQ) [111]. In addition, Ebert et al. showed that PON2-knockout mice presented loss of redox homeostasis, endothelial cells’ abnormalities with an increase of tissue factor activity, reduction of coagulation times, and increased platelet activity [112].

#### 5.1.2. NADPH Oxidase

This multisubunit enzyme is also reported to promote ROS generation in the endothelium. Wu et al. showed that, in microvascular endothelial cells when stimulated with LPS, the ROS scavenger ascorbate abrogated NOX1 activity; p47phox expression; and, consequently, decreased ROS production [113]. The upregulation of NOX1 by LPS, TNF-α, and IL-1α has also been shown to induce mitochondrial O_2_^•−^ generation in pig pulmonary arteries [114].

During sepsis, the endothelium generates high levels of tissue factor pathway inhibitor (TFPI), together with NO and prostacyclin, in order to maintain an antithrombotic capacity. However, in sepsis, endothelium anticoagulant factors such as TFPI do not operate appropriately, thereby allowing leukocyte and platelet adhesion and, consequently, the release of tissue factor and the formation of microthrombi [87,111]. Interestingly, incubation of endothelial cells with xanthine/xanthine oxidase a potent source of O_2_^•−^ and H_2_O_2_, inhibiting TFPI [115].

#### 5.1.3. iNOS and NO

The glycocalyx can also release toxic levels of NO via iNOS activity, which causes hypotension and circulatory failure, subsequent disruption of oxygen distribution impairment of the endothelial barrier system, and damage to various organs [77,116]. Additionally, greatly elevated levels of NO promote a substantial inhibition of platelet activity, leading to an extensive bleeding time, hemorrhage, and potentially death [117,118]. Furthermore, in addition to endothelial cells, neutrophils also contain iNOS, which further contributes to the NO pool in sepsis and reduces the interaction between neutrophils and the endothelium [119]. In this regard, a study using a cecal ligation and puncture (CLP) model of sepsis in rodents showed that increases in NO production inhibited neutrophil rolling and, consequently, abolished adhesion to the endothelium. Importantly, treatment with aminoguanidine, an NO inhibitor, was able to restore neutrophil function and decreased mortality [120]. Another relevant pathophysiological RNS species is ONOO^−^, which is formed by the reaction between equimolar levels of O_2_^•-^ and NO; this directly damaging species when formed is associated with cytotoxic effects and tissue damage [77,116,121].

### 5.2. Platelets

Platelets also play a key role in ROS production during sepsis, with both external and internal ROS production reported to modulate platelet activity through the integrin αIIbβ_3_ (fibrinogen receptor), GVI (collagen receptor), and GPIbα (von Willebrand factor receptor) [122,123]. As such, platelets incubated with LPS from *Chlamydia pneumoniae*, *Proteus mirabilis*, or *Escherichia coli* all demonstrated elevated levels of ROS generation. In addition, LPS induced ROS generation by platelets and increased platelet–fibrinogen binding and P-selectin exposure, all of which was abrogated by superoxide dismutase and catalase [107,122]. Moreover, mice pre-treated with the antioxidant *N*-acetylcysteine (NAC) prior to injection with LPS re-established normal levels of platelet ROS production and aggregation [124].

One of the main roles of platelets in sepsis is to promote the activation and migration of neutrophils to the sites of tissue injury and to stimulate neutrophil NETs’ release and ROS generation [125,126]. Additionally, both in experimental sepsis and in patients recovering from septic shock, neutrophils also generate extensive levels of production ROS via NADPH oxidase (NOX2), independent of any platelet stimulus. Importantly, neutrophils’ surface receptors, such as integrin, Fc receptors, and members of the G-protein-coupled receptors family, have all been shown to promote the stimulation of neutrophil-NADPH oxidase activation [127].

It is well known that platelets contain NADPH oxidases, more specifically NOX1, NOX2, and NOX4. However, any role of NOXs in platelet signalling remains somewhat contradictory [128,129,130,131]. Some studies have reported a fundamental role in experimental platelet ROS production, while others have shown that endotoxemia in rats increased TNF-α levels and promoted platelet-NADPH oxidase activity, via cGMP-PKG and PKC-p47phox signalling pathways [22,124,132]. NADPH oxidase (NOX2) activity within neutrophils and specifically ROS production has been suggested to stimulate NETosis, as seen in both human and experimental sepsis [104,133]. However, this assertion is a subject of ongoing debate, as other studies have shown that inhibition of neutrophil NADPH oxidase did not affect NETs’ release [134,135].

Moreover, in patients with sepsis, soluble plasma factor-induced uncoupling of platelet mitochondria increases respiratory capacity, a feature that was more intense in non-survivors. In addition, platelet mitochondria function was reported to be associated with organ failure and elevated lactate levels [136,137].

## 6. Conclusions

This review provides some evidence linking aspects of oxidative/nitrosative stress, and the onset and establishment of hemostasis in sepsis. Abnormal coagulation events including DIC impair tissue prefusion, which may ultimately result in multiple organ dysfunction and death. The combination of greatly elevated levels of ROS and RNS production resulting from an overstimulation of the inflammatory response beyond the limits of homeostatic control, in part due to depletion of finite antioxidant reserves, results in a pro-oxidant environment. Under these circumstances, redox-based stress responses become detrimental, with an array of negative impacts including the over-stimulation of coagulation and further expansive inflammatory systems’ activation; endothelium dysfunction and platelet and neutrophil activation, including the formation of neutrophil NETs, all contribute to these responses.

**Figure 2:**
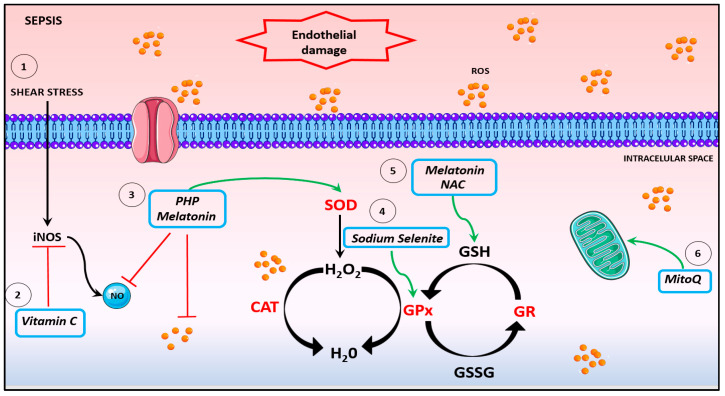
Potential antioxidants therapies. (1) The inflammatory scenario of sepsis induces shear stress, causing endothelium damage and activation of iNOS, leading to an NO boosting. (2) Vitamin C is an antioxidant acting on iNOS inhibition expression, improving microvascular dysfunction and ameliorating hypotension. (3) The compound PHP and melatonin sequestrate NO and promote SOD activation. (4) Sodium selenite promotes an increase in GPx activity. (5). NAC and melatonin restore GSH levels and inhibit platelet and neutrophil dysfunction. (6). MitoQ enhances mitochondrial respiration and restores mitochondrial dysfunction. NO: nitric oxide. CAT: catalase. SOD: superoxide dismutase. GPx: glutathione peroxidase. GR: glutathione reductase.

Diagnosis and treatment of sepsis remain a significant challenge for healthcare providers globally, and gaining greater insights into key aspects of complicated proinflammatory processes that ensue during onset and progression of disease remains a priority. Targeting the drivers of excessive oxidative/nitrosative stress using antioxidant treatments is an obvious therapeutic avenue. However, while beneficial responses can be demonstrated for an array of adverse endpoints including clotting dysfunction using in vitro and in vivo models, clinical trials have to date been somewhat disappointing; a more nuanced approach may offer a way forward. In this regard, the advent of antioxidants that are specific for key compartments/organelles such as mitochondria, combinational approaches to operative in differing extracellular and intracellular compartments, prophylactic usage in risk groups, and/or the timing and/or duration of use may provide some measure of success (see Figure 2 and Table 2).

## Figures and Tables

**Table 1:** Main reactive oxygen and nitric species producers.

Enzyme	Mechanism	Reference
Nicotinamide adenine dinucleotide phosphate (NADPH) oxidase (NOX1-5; DUOX1, 2)	Conversion of O_2_ to O_2_^•−^, NADPH acts as an electron donor. NOX1–4 provide constitutive activity, which is dependent on subunits NOXO1, p47phox, or p22 phox phosphorylation. Further rearrangement of the subunit complexes p40phox, p67phox, and Rac from the cytosol to the membrane allows for transfer of electrons from the substrate to O_2_. NOX5 and Duox activation are calcium-dependent.	[90,91,92]
Mitochondrial respiration chain	Oxygen acts as the terminal electron acceptor of the respiratory chain. The process involves a four-electron reduction of oxygen to water, which can occur in the outer membrane, in the inner membrane, or within the matrix. ROS including O_2_^•−^, H_2_O_2_, and ^•^OH are produced as intermediates in this ongoing process. Around 1% of O_2_^•−^ exits the mitochondria as a physiological process under steady-state conditions. Hyperoxia and hypoxia/reperfusion both augment O_2_^•−^ release greatly, with the potential for direct effects on cellular redox state and signaling, as well as the conversion to more damaging species through iron catalysis (Fenton reaction).	[93,94,95,96,97]
Cyclooxygenase and Lipoxygenase	These enzymes metabolize arachidonic acid (AA) to form prostaglandins, thromboxane, and leukotrienes. The enzymic addition of oxygen as occurs in these processes involves ROS generation with the potential for collateral effects. In addition, COX and LOX metabolites are known to affect intracellular redox balance by activation of NOX enzymes.	[98,99]
Xanthine, oxidoreductase (XO), dehydrogenase oxidase (XDH)	Rate-limiting enzymes responsible for the conversion of hypoxanthine and xanthine to uric acid in the last stages of purine catabolism. XDH catalyses these process, utilising NAD+ as a cofactor. XDH can be readily converted to XO by hyperoxia, by the effects of ischaemia/reperfusion, or by limited proteolysis. XO catalyses the same reaction, but uses oxygen as a co-factor rather than NAD^+^; consequently, O_2_^•−^ and H_2_O_2_ are generated as by products and thus influence an array of ROS-related dysfunctions.	[71,100,101]
Nitric oxide synthase (NOS) NOS1 or nNOS (neuronal), NOS2 or iNOS (inducible), and NOS3 or eNOS (endothelial)	Enzymatic production of NO and regulation of vascular tone. Use of l-arginine and O_2_ as substrates and nicotinamide-adenine-dinucleotide phosphate (NADPH), flavin adenine dinucleotide (FAD), flavin mononucleotide (FMN), and (6R)5,6,7,8-tetrahydrobiopterin (BH4) as reduced cofactors. When NO is produced by endothelial cells, it diffuses through smooth muscle cells, binding to guanylyl cyclase (GC). GC produces the second messenger, cyclic guanosine 3,5-monophosphate (cGMP). cGMP interacts with protein kinase G (PKG), which promotes the phosphorylation of contractile proteins, resulting in a decrease in cytosolic Ca^2+^, which stimulates myosin light-chain dephosphorylation, promoting vasorelaxation. Formed by the reaction between equimolar amounts NO and O_2_^•−^, the peroxnitrite ion (ONOO^−^) is more reactive and toxic than NO. It modifies proteins and peptides via nitration (of tyrosine) and nitrosylation (of thiol moieties) and, in addition, via hydroxylation reactions involving a species likened to ^•^OH. ONOO^−^ formation and damage is strongly correlated with a range of cardiovascular pathologies.	[101]

**Table 2:** Potential antioxidant therapies.

Therapy	Mechanism	Positive Effect	Why is Not it Been Clinically Used?
Vitamin C	Potent ROS scavenging antioxidant agent [138]	Septic shock patients treated with ANON^®^, an antioxidant-enriched concentrated liquid diet with high concentrations of vitamin C and E, demonstrated a restoration of vitamin C radical levels in serum and a reduction in MOF [139]. Septic animals treated with vitamin C showed an improvement in microvascular dysfunction and microvascular permeability barrier integrity, inhibition of iNOS expression, and ameliorated hypotension [89,113,138,140]. The vasodilatation and reduction in vitamin C plasma concentration after low doses of LPS administration in healthy volunteers were reversed by co-administration of vitamin C [141].	Limited clinical trials
Seleniun	A micronutrient fundamental for GPx synthesis [142,143]	The administration of high levels of sodium selenite intravenously showed an increase in blood selenium concentration and GPx activity and significantly decreased mortality of septic patients with DIC [144].	Seleniun decreased the infection in nonseptic patients only. Clinical trials did not show any improvement in outcomes in a general septic patient population [145]
*N*-acetylcysteine (NAC)	Antioxidant is able to restore the levels of GSH in the cells and also acts as an anti-inflammatory agent [146]	The treatment of rats with NAC, 30 min after LPS injection, re-established their ROS generation levels and platelet aggregation [124]. NAC treatment in rats decreased neutrophil infiltration and leukocyte adherence, ameliorated mitochondrial dysfunction, and decreased oxidative stress [147,148,149,150]. NAC administration by septic patients reduced lipid peroxidation, induced tissue oxygenation, ameliorated cardiac function, and decreased the mortality rate [151,152,153,154].	Conflicting results: some studies showed that NAC did not improve outcome for patients or affect levels of cytokines’ release [155]. NAC can also worsen organ failure [156]. Findings need to be confirmed in larger clinical trials
MitoQ	Targets mitochondrial dysfunction [157]	Endotoxemic rats that received MitoQ by i.v. administration demonstrated enhancement in mitochondria respiration, decreased levels of oxidative stress and IL-6, and improved organ dysfunction [157,158].	There are no data from human studies
Superoxide dismutase (SOD)	Converts superoxide radical into hydrogen peroxide and molecular oxygen (O_2_), while the catalase and peroxidases convert hydrogen peroxide into water [159,160]	The M40401 SOD mimetic restored vascular reactivity, regulated arterial pressure, and decreased mortality levels of rats infected with *E. coli* [161]	There are no data from human studies
Nitric oxide scavenger	The compound pyridoxylated haemoglobin polyoxyethylene (PHP) is a chemically altered human-derived hemoglobin used as an NO scavenger and SOD mimetic [162].	In a *Pseudomona aeruginosa* sepsis model in sheep, infusion of PHP for 48 h restored a low mean arterial pressure and improved the systemic vascular resistance [163,164]. In phase I/II clinical trials, PHP increased blood pressure and diminished catecholamine requirement [165]; in a phase III trial with 377 patients, PHP reduced the necessity of vasopressor use [166].	Despite some positive results, after 28 days of therapy with PHP, there was no benefit and indeed mortality rates increased, with a SOFA score higher than 13 [166]
Melatonin	Secreted during the night, melatonin is a hormone produced by the pineal gland. It possesses anti-inflammatory properties and demonstrates antioxidant functions, acting as both an ROS and RNS scavenger [167].	In septic rats induced by CLP, administration of melatonin improved organ injury; an effect that was ascribed to the capacity of melatonin to enhance GSH levels and to inhibit neutrophil aggregation [168]. In a placebo-controlled study with 12 healthy volunteers, the group that received melatonin before LPS showed lower levels of inflammatory markers and oxidative stress compared with the saline control group [138,169].	Lack of clinical trials
